# Association between marital status and mortality risk in cardiac disease: a cardiopulmonary exercise testing cohort study

**DOI:** 10.1265/ehpm.25-00433

**Published:** 2026-03-31

**Authors:** Shinya Takahashi, Atsuko Nakayama, Mamoru Nanasato, Mitsuaki Isobe

**Affiliations:** 1 Department of Cardiology, Sakakibara Heart Institute, Tokyo, Japan; 2 Department of Cardiovascular Medicine, National Hospital Organization Takasaki General Medical Center, Gunma, Japan

**Keywords:** Marital status, Social determinants of health, Cardiopulmonary exercise testing, Cardiac disease, Mortality

## Abstract

**Background:**

Although marital status is a key social determinant of health, its prognostic relevance in cardiopulmonary exercise testing (CPX) cohorts remains unclear. We aimed to evaluate the association between marital status and mortality risk in patients with cardiac disease (CD) who underwent CPX.

**Methods:**

This retrospective, single-center observational study involved consecutive patients with CD who underwent post-discharge CPX between 2008 and 2020. Participants (mean age: 69 years; 73% male) were categorized as either unmarried (never married, divorced, or widowed) or married. The primary outcome was all-cause mortality, and the secondary outcome was cardiovascular mortality. We used Cox proportional hazards models to estimate adjusted hazard ratios (aHRs) and 95% confidence intervals (CIs). Model 1 was adjusted for age and sex to estimate the overall association between marital status and outcomes. Model 2 was adjusted for Model 1 covariates and peak VO_2_ to account for objectively measured exercise capacity.

**Results:**

Of 4,681 patients analyzed, 1,117 were unmarried and 3,564 were married. In Model 1, being married was associated with a lower risk of all-cause mortality (aHR: 0.75, 95% CI: 0.62–0.91, P < 0.001). This association persisted after adjusting for peak VO_2_ in Model 2 (aHR: 0.79, 95% CI: 0.65–0.96, P = 0.002). For cardiovascular mortality, the estimates were consistent in direction (Model 1; aHR: 0.64, 95% CI: 0.44–0.93, P = 0.019, Model 2; aHR: 0.69, 95% CI: 0.47–1.02, P = 0.061).

**Conclusions:**

In a large CPX cohort of patients with CD, married status was associated with a lower risk of all-cause mortality. The association was attenuated but remained after adjustment for peak VO_2_, suggesting that differences in exercise capacity may contribute but do not fully account for the observed association. Marital status should be interpreted as a social marker rather than a causal or interventional exposure, and future studies should clarify modifiable factors related to prognosis.

**Supplementary information:**

The online version contains supplementary material available at https://doi.org/10.1265/ehpm.25-00433.

## Graphical Abstract

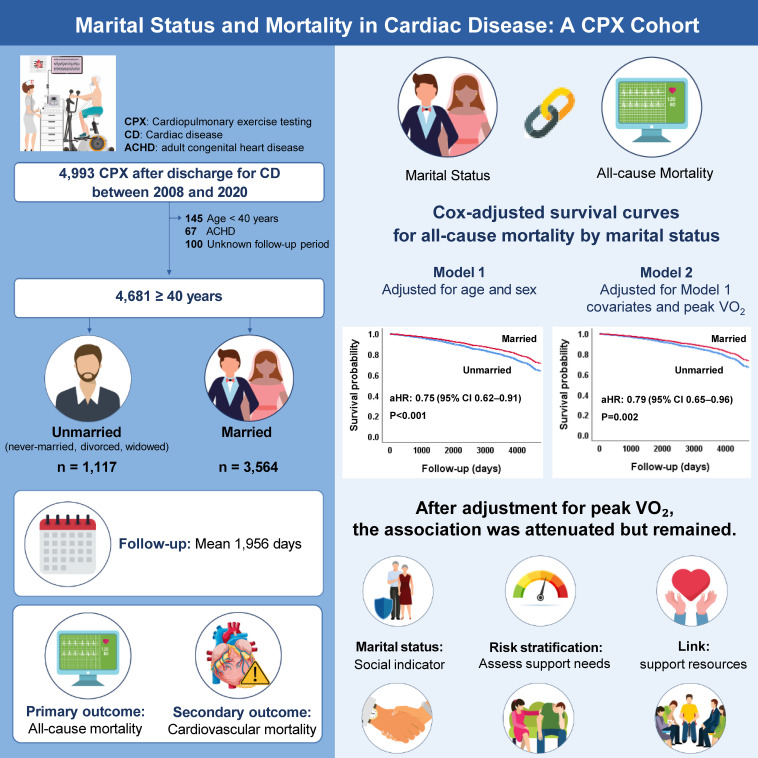

## Background

Marital status in Japan has shifted substantially over the past 35 years. According to a report by the Gender Equality Bureau of the Cabinet Office [1], in 1985, the marriage rate at the age of 40 years was approximately 90% for both men and women. By 2020, this rate had dropped substantially to 65.9% for men and 72.5% for women. Additionally, as a large proportion of marriages among individuals aged 40 years or above are remarriages, the marriage rate at 65 years of age increased only slightly, reaching 76.8% for men and 75.1% for women. The composition of unmarried individuals varies by age: never-married individuals account for more than half of all unmarried individuals under 65 years of age, whereas divorced and widowed individuals account for the majority of those over 65 years of age.

Marital status is a key social indicator. Studies on healthy individuals have shown that unmarried people—including widowed, divorced, and never-married individuals—experience a higher mortality risk than married individuals, regardless of sex [2–4]. A prospective study of 94,062 healthy Japanese citizens demonstrated that unmarried men had a higher risk of mortality than married men. In contrast, the difference in mortality risk based on marital status was not significant for women [5]. Additionally, an association has been observed between cardiovascular mortality risk and marital status in healthy populations [5, 6].

Among patients with cardiac disease (CD), evidence regarding marital status and prognosis is suggestive but remains inconsistent across studies, with some reporting higher mortality in unmarried men and women [7–9], and others reporting an association only among men [10]. In Japan, nationwide claims data indicate that the incidence of CD is substantially higher in men than in women [11]; however, the prognostic implications of marital status in patients with CD have not been well characterized, particularly using objective assessments of functional capacity.

Cardiorespiratory fitness is a strong determinant of prognosis in both healthy individuals and patients with CD, and peak oxygen uptake (peak VO_2_) derived from cardiopulmonary exercise testing (CPX) provides an objective and clinically meaningful measure of exercise capacity [12]. Yet, peak VO_2_ has rarely been incorporated into studies examining marital status and outcomes, leaving uncertainty as to whether social differences in prognosis may be partly accounted for by differences in objectively measured functional capacity. This CPX-based characterization provides a unique opportunity to examine social factors in relation to prognosis on the basis of an objective benchmark of functional capacity.

Therefore, we aimed to examine the association between marital status and mortality in patients with CD who underwent CPX, while accounting for age and sex, and to assess the extent to which the observed association may be partly explained by peak VO_2_. To address this, we evaluated the association with and without adjustment for peak VO_2_.

## Methods

### Study design

For this retrospective, single-center cohort study, we enrolled consecutive patients hospitalized for CD who underwent CPX after discharge as part of routine clinical assessment, provided they were clinically stable and deemed suitable for exercise testing by the attending physician. From 4,993 patients treated at Sakakibara Heart Institute (Tokyo, Japan) between December 2008 and August 2020, we excluded patients aged <40 years, those with unknown follow-up period, and those with adult congenital heart disease. We excluded individuals younger than 40 years to reduce exposure misclassification (marital status is more labile before midlife) and improve cohort homogeneity, as population data [1] indicate a negligible change in marital status distribution after 40 years of age. The final analytic cohort comprised 4,681 patients (mean age 69 years; 73% men).

Marital status and family composition of the patients were assessed using a self-administered questionnaire. The participants were categorized according to marital status as unmarried (never-married, divorced, or widowed) or married (currently having a spouse). Adult child was defined as a child aged ≥18 years. The participants were categorized according to smoking history as former or current smokers. Estimated glomerular filtration rate (eGFR) was calculated using the Japanese equation [13]. CD diagnoses were categorized as follows: ischemic heart disease (IHD), defined as inpatient percutaneous coronary intervention or coronary artery bypass grafting; valvular disease (VD), defined as inpatient valve surgery; congestive heart failure (CHF), defined as hospitalization for heart failure; aortic disease, defined as hospitalization for dissection or aneurysm; and other CDs, including cardiomyopathy, infective endocarditis, and myocarditis.

### CPX protocol

For CPX, we used a symptom-limited, ramp-incremental protocol on an electronically braked cycle ergometer. Two systems were used: ML-9000 (Fukuda Denshi, Tokyo) through 2012 and MLX-1000 (Fukuda Denshi, Tokyo) from 2013 onward. Expired gases were analyzed breath-by-breath with a mass-spectrometry system (AERO MONITOR AS-300S; Minato Medical, Tokyo, Japan). The tests proceeded to volitional exhaustion or limiting symptoms, in accordance with CPX guidelines [14, 15]. Peak VO_2_ was defined as the highest 30-s averaged VO_2_ obtained during exercise. Because peak VO_2_ is effort dependent, we used the respiratory exchange ratio (RER) as an effort indicator, and peak effort was considered achieved when RER was ≥1.10. %peak VO_2_ was calculated as measured peak VO_2_ divided by predicted peak VO_2_ derived from the age- and sex-specific reference equations reported by Itoh et al. [16].

### Outcomes and follow-up

The primary outcome was all-cause mortality; cardiovascular mortality was the secondary outcome and was defined a priori as death due to fatal myocardial infarction, ischemic stroke, sudden cardiac death, or multiorgan failure of cardiac origin. Outcomes were ascertained from the index CPX through the last follow-up (mean: 1,956 days, interquartile range [IQR]: 990–3,141 days), with censoring at the earlier of the last confirmed contact (clinic visit or telephone) or on April 7, 2024. Vital status and cause of death were determined from medical records and telephone interviews.

This study was approved by the Sakakibara Heart Institute Ethics Committee (Approval No. 16-005). Given the minimal-risk, retrospective design using existing records, the requirement for informed consent was waived and an opt-out notice was provided. The procedures adhered to the tenets of the Declaration of Helsinki and relevant national guidelines.

### Conceptual framework and covariate selection

Marital status was treated as the exposure, and age and sex were included as key baseline covariates. Exercise capacity (peak VO_2_) was incorporated as an objective measure of functional status that may partly account for differences in prognosis across social groups. To minimize potential overadjustment and because several clinical and behavioral factors may be correlated with marital status, variables such as smoking history, BMI (body mass index), and baseline clinical comorbidities were not included in the primary models and were examined only in sensitivity analyses. Subtypes of CD diagnosis were reserved for prespecified subgroup analyses and were not included as adjustment covariates in the primary models. Marital status was ascertained at baseline and treated as time-fixed.

### Statistical analysis

Continuous variables are summarized as median (IQR) and categorical variables as count (%). Between-group comparisons were performed using the Mann–Whitney U test and chi-square test, as appropriate. We fitted Cox proportional hazards models for the primary outcome (all-cause mortality) and the secondary outcome (cardiovascular mortality), using time from the index CPX as the time scale and marital status (married vs. unmarried) as the exposure of interest. Model 1 was adjusted for age and sex to estimate the overall association between marital status and outcomes. Model 2 was adjusted for Model 1 covariates and peak VO_2_ to account for objectively measured exercise capacity. To visualize absolute risk, we plotted Cox-adjusted survival curves for all-cause mortality by marital status based on Models 1 and 2. For display purposes only, the x-axis was truncated at 4,500 days because the number at risk became small thereafter; this did not affect estimation of hazard ratios or adjusted survival functions.

Analytic procedures were prespecified, including sensitivity analyses, assessment of functional form using restricted cubic splines, evaluation of time-varying effects, and prespecified subgroup and interaction analyses. Sensitivity analyses included: (i) further adjustment to Model 2 for BMI, smoking, and baseline clinical comorbidities to evaluate whether the findings were robust to additional clinical risk adjustment; and (ii) a 30-day landmark analysis for Models 1 and 2, restricting the risk set to patients who were alive and event-free at 30 days after the index CPX. Linearity and functional form were evaluated using restricted cubic splines. Overall associations and nonlinearity were examined using likelihood-ratio tests (overall: variable excluded vs. full spline model; nonlinearity: linear-only vs. linear + spline). Proportional hazards assumptions were assessed using extended Cox models with covariate × ln(time) terms and a global likelihood-ratio (omnibus) test. The spline and time-varying assessments were prespecified for the primary outcome (all-cause mortality) only. For cardiovascular mortality, we fit cause-specific Cox models (treating non-cardiovascular deaths as censored), rather than Fine–Gray subdistribution models, as cumulative incidence was not the primary estimand. Prespecified subgroups included age group (40–64 vs. ≥65 years; used for subgroup analyses only), sex, adult-child status (none vs. ≥1), and CD diagnosis (IHD, VD, and CHF). The 65-year threshold was chosen a priori based on epidemiologic data indicating an age-related shift in the composition of the unmarried population in Japan [1] and to provide a clinically interpretable subgroup comparison. Aortic disease and other cardiac conditions were excluded from multivariable models owing to low events per variable. Within each subgroup, models followed Model 2 for all-cause mortality, except that the subgroup-defining variable(s) were omitted from the linear predictor. For age-stratified analyses, age was retained as a continuous covariate within each stratum. Interactions were tested using two-sided likelihood-ratio tests (α = 0.05).

As variable-level missingness was low (<5%), we used complete-case analysis. Counts of missing values for each variable are reported in the footnote to Table 1 (percentages use variable-specific available denominators). Two-sided P < 0.05 was considered statistically significant. Analyses were performed using SPSS, version 26 (IBM, Armonk, NY, USA). This observational cohort study followed the STROBE guidelines.

**Table 1:** Baseline characteristics

**Characteristic**	**Unmarried** **(n = 1117)**	**Married** **(n = 3564)**	**P value**
Age (years), median (IQR)	71 (59–77)	69 (61–76)	0.023
Age ≥65 years	745 (67)	2370 (66)	0.901
Male individuals, n (%)	607 (54)	2830 (79)	<0.001
Having an adult child, n (%)	624 (56)	2316 (65)	<0.001
BMI (kg/m^2^), median (IQR)	23.0 (21.0–25.7)	23.2 (21.3–25.1)	0.936
Smoking history, n (%)	488 (46)	1670 (49)	0.091
Family history of CD, n (%)	91 (8.3)	335 (9.5)	0.621
**Comorbidity**			
Hypertension, n (%)	749 (67)	2368 (67)	0.975
Dyslipidemia, n (%)	718 (64)	2394 (67)	0.074
Diabetes mellitus, n (%)	406 (36)	1334 (37)	0.514
Atrial fibrillation, n (%)	136 (12)	334 (9.3)	0.007
**Cardiac Disease**			
Ischemic heart disease, n (%)	562 (50)	2025 (57)	<0.001
Valvular disease, n (%)	248 (22)	681 (19)	0.024
Congestive heart failure, n (%)	178 (16)	469 (13)	0.019
Aortic disease, n (%)	63 (5.6)	221 (6.2)	0.493
Other CDs, n (%)	66 (5.9)	168 (4.7)	0.110
**Device**			
ICD, n (%)	52 (4.6)	179 (5.0)	0.621
**Medication use**			
ACE-i or ARB, n (%)	499 (45)	1558 (44)	0.484
Beta-blocker, n (%)	667 (60)	2055 (58)	0.448
MRA, n (%)	144 (13)	457 (13)	0.830
Statin, n (%)	500 (45)	1666 (47)	0.280
**Blood test**			
Hemoglobin (g/dL), median (IQR)	12.8 (11.7–14.0)	13.2 (12.1–14.2)	<0.001
Serum creatinine (mg/dL), median (IQR)	0.82 (0.70–1.03)	0.90 (0.76–1.06)	<0.001
eGFR (mL/min/1.73 m^2^), median (IQR)	62 (50–72)	63 (52–72)	0.484
Triglyceride (mg/dL), median (IQR)	139 (97–187)	121 (91–190)	0.857
LDL-cholesterol (mg/dL), median (IQR)	99 (76–126)	92 (72–111)	0.039
HDL-cholesterol (mg/dL), median (IQR)	48 (40–60)	49 (42–58)	<0.001
hs-CRP (mg/dL), median (IQR)	0.06 (0.02–0.16)	0.06 (0.02–0.19)	0.637
**Cardiopulmonary exercise testing**			
Peak VO_2_ (mL/kg/min), median (IQR)	16.5 (13.5–19.7)	17.6 (14.4–21.3)	<0.001
%peak VO_2_ (%), median (IQR)	70 (58–85)	75 (63–89)	<0.001

Note: Data are presented as n (%) unless otherwise indicated. Percentages use variable-specific available denominators. IQR: interquartile range, BMI: body mass index, ICD: implantable cardioverter-defibrillator, ACE-i: angiotensin-converting enzyme inhibitor, ARB: angiotensin receptor blocker, MRA: mineralocorticoid receptor antagonist, eGFR: estimated glomerular filtration rate, LDL: low-density lipoprotein, HDL: high-density lipoprotein, hs-CRP: high-sensitivity C-reactive protein, Peak VO_2_: peak oxygen uptake. Missing data (overall): smoking history 210 (4.5%), family history of CD 58 (1.2%), ACE-i or ARB use 28 (0.6%), beta-blocker use 28 (0.6%), MRA use 28 (0.6%), statin use 28 (0.6%), hemoglobin level 95 (2.0%), serum creatinine level (mg/dL) 107 (2.3%), eGFR (mL/min/1.73 m^2^) 107 (2.3%), triglyceride level (mg/dL) 164 (3.5%), LDL-cholesterol level (mg/dL) 185 (4.0%), HDL-cholesterol level (mg/dL) 200 (4.3%), and hs-CRP level (mg/dL) 175 (3.7%). Denominators shown per variable.

## Results

### Flowchart of patient enrolment

**Fig. 1:**
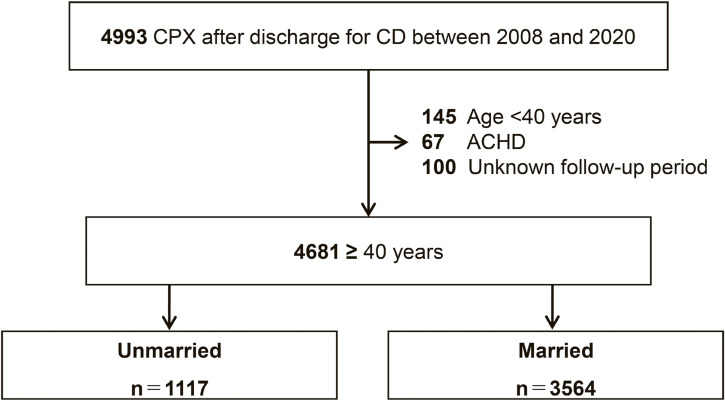
Flowchart of patient enrolment. Note: CPX: cardiopulmonary exercise testing, CD: cardiac disease, ACHD: adult congenital heart disease. Patient inclusion/exclusion from the initial cohort to the final analytic sample.

Figure 1 presents the flowchart of patient enrolment. Between 2008 and 2020, 4,993 patients underwent CPX after discharge following hospitalization for CD. After excluding 145 patients aged <40 years, 100 with unknown follow-up period and 67 with adult congenital heart disease, 4,681 patients were included. According to the marital status at the index CPX, 1,117 patients were classified as unmarried and 3,564 as married.

### Baseline characteristics

Table 1 shows the baseline characteristics of the patients. At baseline, the unmarried group was significantly older than the married group (P = 0.023). The married group had a higher percentage of men (P < 0.001) and was more likely to have at least one child than the unmarried group (P < 0.001). Atrial fibrillation (P = 0.007) was more prevalent in the unmarried group than in the married group. Cardiac diagnoses differed: IHD was more prevalent in the married group than in the unmarried group (P < 0.001), whereas VD (P = 0.024) and CHF (P = 0.019) were more prevalent in the unmarried group than in the married group. The married group had higher hemoglobin and creatinine (both P < 0.001), lower LDL cholesterol (P = 0.039), and higher HDL cholesterol levels (P < 0.001) than the unmarried group. Exercise capacity was higher in the married group than in the unmarried group (peak VO_2_ and %peak VO_2_, both P < 0.001). There were no significant differences in other characteristics between the groups.

### Outcomes

Table 2 shows the outcomes. For the primary outcome of all-cause mortality, there were 165 events in the unmarried group and 444 in the married group (P = 0.045). For the secondary outcome of cardiovascular mortality, there were 42 and 105 events in the unmarried and married groups, respectively (P = 0.174).

**Table 2:** Outcomes

**Outcome**			

	**Unmarried** **(n = 1117)**	**Married** **(n = 3564)**	**P value**
All-cause mortality, n (%)	165 (15)	444 (12)	0.045
Cardiovascular mortality, n (%)	42 (3.7)	105 (2.9)	0.174

### Cox regression analyses

Table 3 shows the results of the Cox regression analyses.

**Table 3:** Cox regression analyses

	**HR**	**95% CI**	**P value**
**Univariate analysis**			
All-cause mortality			
Married	0.82	0.69–0.98	0.033
Cardiovascular mortality			
Married	0.76	0.53–1.08	0.124

**Model 1: adjusted for age and sex**			
All-cause mortality			
Married	0.75	0.62–0.91	<0.001
Age (per 1-year increase)	1.07	1.06–1.08	<0.001
Sex (male)	1.90	1.54–2.34	<0.001
Cardiovascular mortality			
Married	0.64	0.44–0.93	0.019
Age (per 1-year increase)	1.04	1.02–1.06	<0.001
Sex (male)	2.12	1.36–3.30	<0.001

**Model 2: adjusted for Model 1 covariates and peak VO_2_.**			
All-cause mortality			
Married	0.79	0.65–0.96	0.002
Age (per 1-year increase)	1.04	1.03–1.05	<0.001
Sex (male)	2.54	2.05–3.14	<0.001
Peak VO_2_ (per 1 mL/kg/min increase)	0.85	0.83–0.87	<0.001
Cardiovascular mortality			
Married	0.69	0.47–1.02	0.061
Age (per 1-year increase)	1.02	1.01–1.04	0.042
Sex (male)	3.18	2.00–5.05	<0.001
Peak VO_2_ (per 1 mL/kg/min increase)	0.83	0.80–0.87	<0.001

In the univariate analysis, being married was associated with a significantly lower risk of all-cause mortality (hazard ratio [HR]: 0.82, 95% confidence intervals [CI] 0.69–0.98, P = 0.033), whereas the association with cardiovascular mortality did not reach statistical significance (HR: 0.76, 95% CI: 0.53–1.08, P = 0.124).

In Model 1 (adjusted for age and sex), being married was associated with a significantly lower risk of all-cause mortality (adjusted HR [aHR]: 0.75, 95% CI: 0.62–0.91; P < 0.001) and cardiovascular mortality (aHR, 0.64; 95% CI, 0.44–0.93, P = 0.019). Age (per 1-year increase: aHR: 1.07, 95% CI: 1.06–1.08, P < 0.001) and male sex (aHR: 1.90, 95% CI: 1.54–2.34, P < 0.001) were also significantly associated with higher all-cause mortality. Similarly, age (per1-year increase: aHR: 1.04, 95% CI: 1.02–1.06, P < 0.001) and male sex (aHR: 2.12, 95% CI: 1.36–3.30, P < 0.001) were significant predictors of cardiovascular mortality.

In Model 2 (adjusted for Model 1 covariates and peak VO_2_), being married remained significantly associated with a lower risk of all-cause mortality (aHR: 0.79, 95% CI: 0.65–0.96, P = 0.002). Age (per 1-year increase: aHR: 1.04, 95% CI: 1.03–1.05, P < 0.001) and male sex (aHR: 2.54, 95% CI: 2.05–3.14, P < 0.001) were associated with higher risk, whereas higher peak VO_2_ was associated with a lower risk (per 1 mL/kg/min increase: aHR: 0.85, 95% CI: 0.83–0.87, P < 0.001). For cardiovascular mortality, being married showed a borderline association (aHR: 0.69, 95% CI: 0.47–1.02, P = 0.061). Age (per 1-year increase: aHR: 1.02, 95% CI: 1.01–1.04, P = 0.042) and male sex (aHR: 3.18, 95% CI: 2.00–5.05, P < 0.001) were associated with a significantly higher risk, whereas higher peak VO_2_ was associated with a significantly lower risk (per 1 mL/kg/min increase: aHR: 0.83, 95% CI: 0.80–0.87, P < 0.001).

**Fig. 2:**
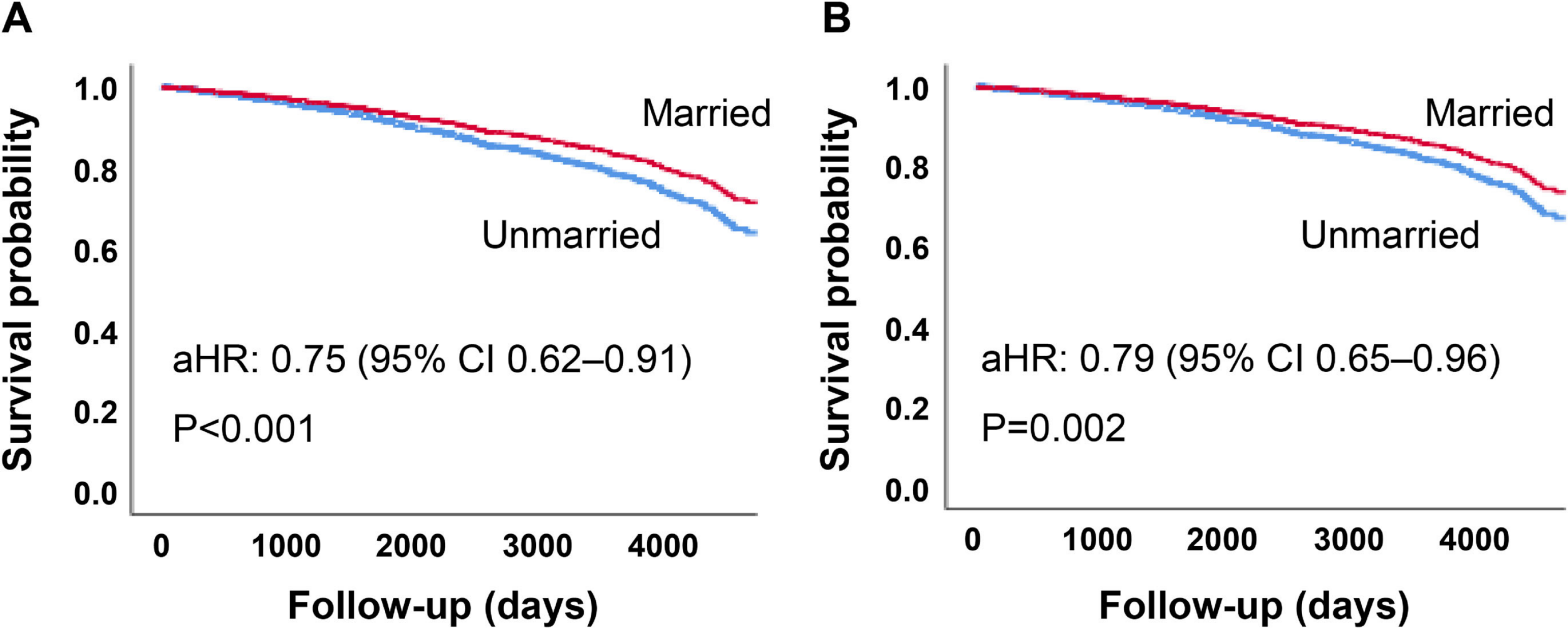
Cox-adjusted survival curves for all-cause mortality by marital status. Note: aHR: adjusted hazard ratio, CI: confidence interval. (A) Model 1 was adjusted for age and sex to estimate the overall association between marital status and outcomes. (B) Model 2 was adjusted for Model 1 covariates and peak VO_2_ to account for objectively measured exercise capacity. For visibility, the x-axis is truncated at 4,500 days because the number at risk becomes small thereafter; estimates beyond this point should be interpreted cautiously.

Figure 2 shows Cox-adjusted survival curves for all-cause mortality by marital status. In Model 1 (A), marital status (married vs. unmarried) was associated with an approximately 25% lower risk of all-cause mortality. In Model 2 (B), the association was attenuated after additional adjustment for peak VO_2_ but remained statistically significant.

The findings remained materially unchanged in sensitivity analyses that additionally adjusted for BMI and baseline clinical comorbidities (Additional File 1: Table S1). In the 30-day landmark analysis (n = 4,653), the effect estimates in Models 1 and 2 were directionally consistent and statistically significant. In the primary analyses, age and peak VO_2_ were modeled as a continuous linear covariate. The analyses using restricted cubic splines suggested a nonlinear association for age; however, the main findings were materially unchanged compared with the linear specification. In contrast, peak VO_2_ showed a strong overall association with no evidence of meaningful nonlinearity (Additional File 1: Table S2). Tests for non-proportional hazards were significant overall; however, only the age × ln(time) term was consistently significant. Marital status showed no violation of proportional hazards, and its conclusions remained unchanged in extended Cox models for both outcomes (Additional File 1: Table S3).

### Subgroup analyses

In effect modification, the association between marital status and all-cause mortality differed by age group (P for interaction <0.001), whereas interaction with sex was not significant (P for interaction = 0.857).

**Fig. 3:**
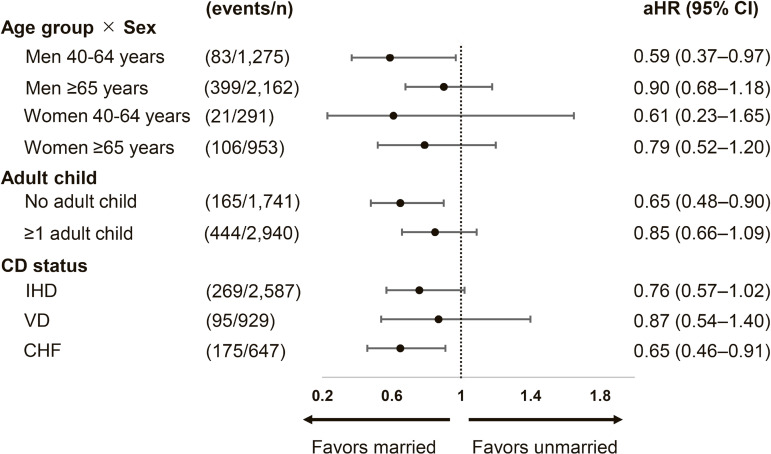
Subgroup analysis of all-cause mortality from Model 2. Note: HR: hazard ratio, CI: confidence interval, IHD: ischemic heart disease, VD: valvular disease, CHF: congestive heart failure, CD: cardiac disease. Points show hazard ratios (solid circles) with 95% CIs; the vertical dashed line indicates HR = 1. Counts to the right of each estimate are shown as events/n. HR < 1 favors the married group. Aortic disease and other CDs were excluded from multivariable modeling owing to low events per variable and unstable estimates.

Subgroup estimates from Model 2 are presented for interpretability (Fig. 3) and should be considered exploratory given multiple comparisons. Overall, the point estimates were generally in the same direction across subgroups (HR < 1 for married vs. unmarried), although most subgroup comparisons did not reach statistical significance. Statistically significant associations were observed in men aged 40–64 years and in participants without adult children. In diagnosis-stratified analyses, a statistically significant association was observed among patients with CHF, whereas associations in other diagnostic subgroups were not statistically significant.

## Discussion

### Main findings

In this large CPX cohort of patients with CD, being married was associated with a lower risk of all-cause mortality after adjustment for age and sex. The association was attenuated but remained after additional adjustment for peak VO_2_, an objective measure of exercise capacity.

A key contribution of this study is the use of a CPX-characterized cohort, which enabled evaluation of marital status in relation to outcomes while incorporating objectively measured exercise capacity (peak VO_2_), a strong prognostic marker that has rarely been included in prior studies on social factors and prognosis. These findings should be interpreted as observational associations, and causal inferences cannot be made.

### Potential mechanisms

Married individuals may be more likely to engage in health-promoting behaviors, including regular exercise, healthy eating, and quality sleep, through encouragement from their spouse and shared responsibility [17, 18]. In our cohort, married participants showed higher exercise capacity at baseline, including higher %peak VO_2_, than unmarried participants. The association between marital status and all-cause mortality persisted after adjustment for peak VO_2_, suggesting that factors beyond exercise capacity may also be relevant.

Marital status may also capture differences in psychosocial stress and social connectedness. Loneliness and chronic stress have been linked to heightened hypothalamic–pituitary–adrenal axis and sympathetic activation, reduced vagal tone, and pro-inflammatory signaling [19–23]. Lower psychosocial stress and greater social support may be associated with reduced allostatic load and systemic inflammation, potentially relating to survival outcomes independent of cardiorespiratory fitness. In addition, individuals with a cohabiting partner may be more likely to participate in health check-ups and outpatient follow-up, maintain persistence with prescribed therapy, recognize clinical deterioration earlier, and seek timely emergency care [24, 25]. These behavioral and healthcare-utilization differences—including health behaviors such as diet quality and sleep regularity—may influence outcomes through mechanisms not directly reflected by peak VO_2_. Finally, marital status is correlated with broader social resources. Prior studies suggest that social support and networks associated with marriage relate to better psychological health and may be associated with survival outcomes [26, 27].

### Age-related heterogeneity and sex considerations

Prior work has suggested that the association between marital status and health outcomes may differ by age, with stronger associations in younger adults than in older adults [9]. In our subgroup analysis, a statistically significant association was observed in middle-aged adults (40–64 years). The interaction between marital status and sex was not significant; therefore, any discussion of sex-specific patterns should be interpreted cautiously.

Several age- and sex-related factors may plausibly underlie heterogeneity. First, never-married status is more prevalent below 65 years [1], which may be accompanied by smaller social networks and less sustained household support. Second, unmarried middle-aged adults—particularly men—have been reported to have higher smoking prevalence and lower utilization of preventive care, potentially contributing to delayed diagnosis and suboptimal management of comorbidities [24, 28]. Third, divorce or widowhood may be associated with increased health risks in some groups, including younger men [29]. Consistent with these observations, weaker social ties have been associated with higher risks of all-cause mortality and fatal coronary heart disease in men [30]. Among women, social security and community welfare systems in Japan may mitigate some disadvantages associated with unmarried status, and the relatively high rate of parent–child cohabitation among divorced women [31] may help maintain support networks. Nevertheless, given the non-significant sex interaction, these interpretations should be considered exploratory.

### Disease-specific subgroups

In subgroup analyses stratified by the index cardiac condition, married status was associated with lower all-cause mortality among patients with CHF. Among patients with IHD, the association did not reach conventional statistical significance, but the estimates were suggestive of a lower mortality risk in the married group. In contrast, no meaningful association was observed in the VD subgroup. These patterns are broadly in line with prior reports linking unmarried status with higher cardiac risk and mortality [8, 9]. For CHF, our findings are compatible with a meta-analysis of 10 studies reporting higher mortality risk among unmarried patients compared with married patients [32], as well as reports suggesting better medication adherence among married patients with CHF [33]. Because CHF outcomes are closely related to adherence, self-management, and engagement with care, differences in social support and healthcare utilization may be clinically relevant correlates. To our knowledge, evidence remains limited regarding postoperative VD in this context; therefore, the null finding in the valvular subgroup should be interpreted cautiously.

### Implications for clinical practice and public health

Although cardiac-specific programs addressing social isolation are limited, community resources that support social connection and care engagement may be available in many settings [34]. From a clinical and public health perspective, marital status should be interpreted not as a causal exposure but as an easily accessible social indicator, and it may serve as a prompt for more detailed assessment of patients’ support needs. In clinical practice, clinicians may consider assessing living arrangements (e.g., living alone), caregiver availability, and social connectedness/disconnection, and exploring linkage to individually tailored resources when needs are identified.

### Strengths and limitations of this study

This study has several strengths, including the large cohort size, objective assessment of exercise capacity using CPX, detailed definition of CD status, and careful long-term follow-up. In addition, our CPX-characterized cohort enabled evaluation of the extent to which exercise capacity may relate to the observed association between marital status and mortality, as indicated by attenuation after adjustment for peak VO_2_.

However, several limitations warrant consideration. First, this retrospective, single-center study conducted in a Japanese population may limit generalizability. Second, selection bias is possible because the cohort consisted of patients who underwent CPX after discharge and were stable enough to perform exercise testing; thus, more severely ill individuals may have been underrepresented. Although peak VO_2_ is an objective and strong prognostic marker, it may not fully capture baseline disease duration or clinical severity, nor eliminate all confounding. Moreover, the temporal ordering between marital status and health-related behaviors may be heterogeneous and potentially bidirectional; therefore, mediation should not be inferred from comparisons with versus without peak VO_2_ adjustment. Third, we could not distinguish subcategories within the unmarried group, and marital status was assessed only at baseline without accounting for transitions or marital quality. Fourth, other social determinants of health (e.g., income, education, occupation, and social networks) were not available, and BNP/NT-proBNP were inconsistently recorded across the study period (typically as either BNP or NT-proBNP), which precluded their inclusion in the primary models; therefore, residual confounding cannot be excluded. In addition, we lacked direct measures of living arrangements and social support (e.g., living alone, cohabitation, caregiver availability, social isolation, or loneliness); therefore, we could not separate the association of marital status from living alone or related aspects of household support. Finally, the cohort included predominantly men with a relatively small number of women, follow-up was approximately 5 years, and we restricted inclusion to adults aged ≥40 years; thus, subgroup estimates and generalizability to women, younger adults, and longer-term prognosis should be interpreted with caution.

## Conclusions

In a large CPX-characterized cohort of patients with cardiac disease, being married was associated with lower all-cause mortality than being unmarried. This association was attenuated but remained after adjustment for peak VO_2_, suggesting that exercise capacity may contribute but does not fully explain the relationship, and residual confounding cannot be excluded. Marital status should be viewed as a non-modifiable social marker rather than a causal or interventional exposure. Incorporating social context may help identify individuals who could benefit from supportive strategies targeting modifiable downstream factors such as social support and engagement with care.

## Abbreviations

BMI: body mass index
CHF: congestive heart failure
CI: confidence interval
CPX: cardiopulmonary exercise testing
CD: cardiac disease
eGFR: Estimated glomerular filtration rate
HR: hazard ratio
IHD: ischemic heart disease
IQR: interquartile range
peak VO_2_: peak oxygen uptake
VD: valvular disease

## Supplementary information

**Additional file 1:** **Table S1.** Sensitivity analyses. **Table S2.** Linearity and functional form: likelihood-ratio tests from restricted cubic spline models. **Table S3.** Extended Cox regression analyses.

## Declarations

### Availability of data and material

De-identified minimal datasets underlying the results are available from the corresponding author on reasonable request, subject to institutional ethics approval and a data transfer agreement, given privacy and ethical considerations.
