# Supplementary material for: Association between marital status and mortality risk in cardiac disease: a cardiopulmonary exercise testing cohort study
Source: Environ Health Prev Med. 2026 Mar 31;31:26. doi: 10.1265/ehpm.25-00433 (PMC13057906; doi:10.1265/ehpm.25-00433)
Supplement: Supplementary file 1 — Additional file 1: Table S1. Sensitivity analyses. Table S2. Linearity and functional form: likelihood-ratio tests from restricted cubic spline models. Table S3. Extended Cox regression analyses. [file ehpm-31-026-s001.docx]

**Additional File 1**

**Table S1. Sensitivity analyses**

|  | **HR** | **95% CI** | **P value** |
| --- | --- | --- | --- |
| All-cause mortality | | | |
| Married | 0.73 | 0.58–0.92 | 0.007 |
| Age (per 1-year increase) | 1.05 | 1.04–1.06 | <0.001 |
| Sex (male) | 2.72 | 2.04–3.62 | <0.001 |
| Peak VO_2_ (per 1 mL/kg/min increase) | 0.86 | 0.84–0.88 | <0.001 |
| Obesity | 0.99 | 0.79–1.24 | 0.930 |
| Smoking | 0.91 | 0.74–1.11 | 0.330 |
| Hypertension | 1.22 | 0.99–1.51 | 0.062 |
| Dyslipidemia | 1.54 | 1.26–1.90 | <0.001 |
| Diabetes mellitus | 1.36 | 1.11–1.66 | 0.003 |
| Atrial fibrillation | 1.07 | 0.82–1.40 | 0.615 |
| ICD | 2.87 | 2.22–3.71 | <0.001 |
| Cardiovascular mortality | | | |
| Married | 0.59 | 0.38–0.92 | 0.020 |
| Age (per 1-year increase) | 1.02 | 1.00–1.04 | 0.090 |
| Sex (male) | 3.05 | 1.67–5.58 | <0.001 |
| Peak VO_2_ (per 1 mL/kg/min increase) | 0.86 | 0.81–0.90 | <0.001 |
| Obesity | 1.17 | 0.75–1.83 | 0.499 |
| Smoking | 0.90 | 0.60–1.36 | 0.611 |
| Hypertension | 1.16 | 0.76–1.78 | 0.503 |
| Dyslipidemia | 1.78 | 1.16–2.73 | <0.001 |
| Diabetes mellitus | 1.36 | 1.11–1.66 | 0.008 |
| Atrial fibrillation | 1.27 | 0.76–2.13 | 0.355 |
| ICD | 4.01 | 2.54–6.45 | <0.001 |

Note: HR: hazard ratio, CI: confidence interval, Peak VO_2_: peak oxygen uptake, ICD: implantable cardioverter-defibrillator

**Table S2. Linearity and functional form: likelihood-ratio tests from restricted cubic spline models**

| **Variable** | **Overall association**  **(LRT P)** | **Nonlinearity**  **(LRT P)** |
| --- | --- | --- |
| Age knots (years) |  |  |
| 53/69/80 | <0.001 | 0.008 |
| Peak VO_2_ knots (mL/kg/min) | | |
| 12.1/17.6/24.6 | <0.001 | 0.944 |

Note: Peak VO_2_: peak oxygen uptake

Age knots: Percentiles from the analytic sample: 10th = 53, 50th = 69, 90th = 80. Peak VO₂ knots: Percentiles from the analytic sample: 10th = 12.1, 50th = 17.6, 90th = 24.6 mL/kg/min).

**Table S3. Extended Cox regression analyses**

| **Covariate** × **ln(time)** | **γ** | **SE** | **P value** | **HR** | **95% CI** |
| --- | --- | --- | --- | --- | --- |
| **Model 1: adjusted for age and sex** | | | | | |
| **All-cause mortality** | | | | | |
| Married × ln(time) | −0.145 | 0.104 | 0.163 | 0.87 | 0.71–1.06 |
| Age × ln(time) | 0.016 | 0.004 | <0.001 | 1.02 | 1.01–1.03 |
| Sex (male) × ln(time) | −0.193 | 0.123 | 0.118 | 0.82 | 0.65–1.05 |
| **Cardiovascular mortality** | | | | | |
| Married × ln(time) | −0.041 | 0.157 | 0.795 | 0.96 | 0.71–1.31 |
| Age × ln(time) | 0.018 | 0.007 | 0.007 | 1.02 | 1.01–1.03 |
| Sex (male) × ln(time) | −0.596 | 0.297 | 0.045 | 0.55 | 0.31–0.99 |
| **Model 2: adjusted for Model 1 covariates plus peak VO₂** | | | | | |
| **All-cause mortality** | | | | | |
| Married × ln(time) | −0.145 | 0.103 | 0.157 | 0.87 | 0.71–1.06 |
| Age × ln(time) | 0.016 | 0.004 | <0.001 | 1.02 | 1.01–1.03 |
| Sex (male) × ln(time) | −0.142 | 0.103 | 0.238 | 0.87 | 0.69–1.10 |
| **Cardiovascular mortality** | | | | | |
| Married × ln(time) | −0.063 | 0.157 | 0.689 | 0.94 | 0.69–1.28 |
| Age × ln(time) | 0.018 | 0.007 | 0.006 | 1.02 | 1.01–1.03 |
| Sex (male) × ln(time) | −0.517 | 0.287 | 0.072 | 0.60 | 0.34–1.05 |

Note: SE: standard error, HR: hazard ratio, CI: confidence interval, peak VO_2_: peak oxygen uptake

Extended Cox models included all prespecified covariate × ln(time) terms simultaneously (mutual). γ is the coefficient for covariate × ln(time). In Model 2, the time-varying term for peak VO₂ was non-significant in item-wise testing and was therefore not retained in the mutually adjusted model; accordingly, the Peak VO₂ × ln(time) row is omitted. The baseline (non–time-varying) effect of peak VO₂ was retained in Model 2.
